# CHTM1 regulates cancer cell sensitivity to metabolic stress via p38-AIF1 pathway

**DOI:** 10.1186/s13046-019-1253-5

**Published:** 2019-06-20

**Authors:** Mansi Babbar, Ying Huang, Christopher M. Curtiss, M. Saeed Sheikh

**Affiliations:** 1 Department of Pharmacology, SUNY Upstate Medical University, Syracuse, NY 13210 USA; 2 Department of Pathology, State University of New York, Upstate Medical University, Syracuse, NY 13210 USA; 3 Present address: Mansi Babbar, Laboratory of Molecular Gerontology, National Institute on Aging, NIH, Baltimore, MD 21224 USA

**Keywords:** Cancer metabolism, Cell death, CHCHD5, Lung cancer, Metabolic stress, p38, AIF

## Abstract

**Background:**

Recently, we have reported the characterization of a novel protein named Coiled-coil Helix Tumor and Metabolism 1 (CHTM1). CHTM1 localizes to both cytosol and mitochondria. Sequence corresponding to CHTM1 is also annotated in the database as CHCHD5. CHTM1 is deregulated in human breast and colon cancers and its deficiency in human cancer cells leads to defective lipid metabolism and poor growth under glucose/glutamine starvation.

**Methods:**

Human cancer cell lines and tissue specimens were used. CHTM1 knockdown was done via lentiviral approach. CHTM1-expresssion constructs were developed and mutants were generated via site-directed mutagenesis approach. Western blotting, immunostaining, immunohistochemistry, cell fractionation and luciferase assays were performed. Reactive oxygen species and reactive nitrogen species were also measured.

**Results:**

Here we report that CHTM1 deficiency sensitizes human lung cancer cells to metabolic stress-induced cell death mediated by glucose/glutamine deprivation and metformin treatment. CHTM1 interacts with Apoptosis Inducing Factor 1 (AIF1) that is one of the important death inducing molecules. CHTM1 appears to negatively regulate AIF1 by preventing AIF1 translocation to cytosol/nucleus and thereby inhibit AIF1-mediated caspase-independent cell death. Our results also indicate that p38, a stress kinase, plays a critical role in metabolic stress-induced cell death in CHTM1-deficient cells. Furthermore, p38 appears to enhance AIF1 translocation from mitochondria to cytosol particularly in metabolically stressed CHTM1-deficient cells and CHTM1 negatively regulates p38 kinase activity. The expression status of CHTM1 in lung cancer patient samples is also investigated and our results indicate that CHTM1 levels are increased in the majority of lung tumors when compared to their matching normal tissues.

**Conclusion:**

Thus, CHTM1 appears to be an important metabolic marker that regulates cancer cell survival under metabolic stress conditions, and has the potential to be developed as a predictive tumor marker.

**Electronic supplementary material:**

The online version of this article (10.1186/s13046-019-1253-5) contains supplementary material, which is available to authorized users.

## Background

Metabolic reprogramming is one of the key features of cancer [1] which primarily uses glucose and glutamine for energy production and biomass generation [2]. Cancer cells, in the absence of glucose, rely on OXPHOS, glutaminolysis and fatty acid oxidation (FAO) to generate ATP [2]; defects in OXPHOS and FAO affect the growth of cancer cells under glucose/glutamine-deprived conditions [3, 4]. Recently, we have reported the identification and characterization of a novel protein named CHTM1 (Coiled-coil Helix Tumor and Metabolism 1) [5]. Sequence matching that of CHTM1 was also noted in the database as CHCHD5. CHTM1 is a protein of 12.9 kDa localizing in both cytosol and mitochondria [5]. We also determined that CHTM1-deficient cancer cells grew poorly under glucose/glutamine-deficient conditions, while cells with increased exogenous levels of CHTM1 displayed increased growth and survival under same conditions [5]. Our mechanistic studies revealed CHTM1 modulated lipid metabolism to promote cell survival under metabolic stress, and positively regulated the PKC-CREB-PGC-1 alpha signaling axis to regulate expression of genes important for fatty acid oxidation and synthesis [5]. CHTM1 levels were also found to be increased in the majority of human primary colon and breast cancers tested in our study [5]. Thus, our recent report has identified CHTM1 as a novel metabolic marker with altered expression in breast and colon tumors that could be involved in the tumorigenic growth under restricted nutrient supplies.

Lung cancer is the most common cause of cancer-related deaths worldwide [6]. The majority (> 85%) of lung cancers are non-small cell lung carcinoma (NSCLC) type. NSCLCs are associated with significant genetic and cellular heterogeneity [7] and accordingly, the information on a given tumor’s histologic type and the associated molecular changes is becoming increasingly important in planning optimal treatment strategies. For example, identification of EGFR mutations and ALK alterations has led to the development of tyrosine kinase inhibitors (TKIs) to target these abnormalities and their utility in the clinic [8].

Despite recent advances about the molecular pathogenesis NSCLCs, these tumors remain difficult to manage. This is partly because not all NSCLCs harbor similar molecular alterations. Therefore, further studies are needed to identify additional genes/proteins that are altered in lung cancers particularly those that are important for lung cancer growth and survival. Identification of such markers is desirable because of their importance in diagnostics and also as valuable targets for cancer therapeutics.

In our recent study [5] we have reported that CHTM1 is as a novel metabolic marker with altered expression in breast and colon tumors. In the present study, we have also investigated the status and role of CHTM1 in human lung cancer. Here we report a novel function of CHTM1 via which CHTM1 alters lung cancer cell survival under metabolic-stress. For example, CHTM1 interacts with Apoptosis Inducing Factor 1 (AIF1) and affects stress-induced cytosol/nuclear translocation of AIF1 as well as cell death. CHTM1 appears to mediate these effects by negatively regulating the p38 kinase. We have also found that CHTM1 is deregulated in lung cancer patient samples such that it is overexpressed in lung cancer samples when compared to their matching normal tissues. Thus, our study highlights CHTM1 as a novel metabolic marker that is important for the pathophysiology of lung cancer.

## Methods

### Human biological samples

Western blot analyses samples were obtained from a NCI supported network, Cooperative Human Tissue Network. Frozen samples were shipped on dry-ice and kept at − 80 °C for long-term storage. Immunohistochemistry samples were purchased from Biomax (Rockville, MD) as formalin-fixed, paraffin-embedded tissue array slides. Slides were shipped and stored at room temperature. Tissue array slides were performed by a pathologist.

### Antibodies and reagents

We used the following antibodies: anti-HA tag (clone 3F10) (Roche Applied Science), anti-β-actin and anti-alpha-tubulin (Sigma-Aldrich), anti-GAPDH and anti-Vinculin (Santa Cruz), cleaved PARP, pro-caspase 3 and pro-caspase 8, phospho-H2AX, AIF1, p38, phospho-p38, phospho- Hsp27, phospho-MAPKAP2 (Cell Signaling Technologies, Boston, MA), anti-CHCHD4 (Protein Tech, IL), anti-Tim23 (BD Biosciences, San Diego, CA), cytochrome c (Thermofisher Scientific, MA), Smac (Upstate cell signaling, NY). The peroxidase-conjugated anti-rat, anti-rabbit, anti-mouse and anti-goat antibodies were from Vector Laboratories (Burlingame, CA). Rabbit polyclonal antibodies specific for human CHTM1 and CHCM1/Mic25 were produced via ProSci Inc. (Poway, CA) against full-length recombinant protein. For cell transfections, Polyjet and Lipojet (Signagen Laboratories, Rockville, MD) were used. Expression construct sub-cloning was performed using restriction endonucleases from New England BioLabs (Ipswich, MA). p38 inhibitor-SB203580 was from Sigma-Aldrich (St. Louis, MO) and pan-caspase inhibitor- Z-VAD-FMK was from BD Biosciences (San Jose, CA, USA). Other chemical reagents were obtained from Sigma-Aldrich and Thermo Fisher Scientific.

### Cells and culture conditions

The following cell lines were used in this study: HEK293T (human embryonic kidney cells from NIH), MCF-7 (human breast cancer cells from NIH), HeLa (human cervical cancer cells from NIH), A549 (human lung cancer cells from NIH), H1299 (human lung cancer cells from NIH) and H460 (human lung cancer cells from ATCC kindly provided by Dr. Shi Yong Sun, Emory University). Cells were cultured in Dulbecco’s modified Eagle’s medium (DMEM) containing 10% fetal bovine serum (Gemini Bio-Products Inc., West Sacramento, CA). For glucose/glutamine deprivation experiments, cells were washed 3 times with PBS and incubated with DMEM without glucose, glutamine and sodium pyruvate.

### Expression constructs

pCMV6-CHTM1 construct was obtained from Origene, MD, USA. CHTM1 open reading frame was cloned into pSRα-HA-S vector for transient expression and pCEP4 vector for stable expression respectively. GST-tagged CHTM1 was produced with PCR-amplified full-length CHTM1 cDNA into pGEX6P-1 expression vector (GE Healthcare, Pittsburgh, PA, USA). Mutation and deletion constructs were synthesized with quick change site-directed mutagenesis kit (Agilent, Santa Clara, CA) following manufacturer’s protocol. Sequencing was performed to validate the authenticity of expression constructs.

### Luciferase assays

pFA-ATF2 and pFR-Luc (Agilent, Santa Clara, CA) were used to measure p38/ATF2 activity. Cells were transfected with pFR-Luc, pFA-ATF2 and pSRα-HA-S CHTM1 or empty vector in the ratio of 1:0.25:1.Luciferase assays were performed as previously reported [9].

### Lentivirus-mediated shRNA silencing

Endogenous CHTM1 was knockdown by the lentivirus-mediated shRNA approach [5]. The scramble shRNA construct was obtained from Addgene, Inc. (Cambridge, MA, USA). All CHTM1 specific shRNA constructs were obtained from Origene, MD, USA. Three different nucleotide sequences were used to target the human CHTM1: KD-1, 5′-CTTAAGGTAGTGACAGTCC-3′; KD-2, 5′-TCTGTCGAAGACACTCCTC-3′ and KD-3, 5′-TGGAAGTCCTGATATCCAG-3′. Addgene protocol was followed for virus production and infection [5].

### Western blotting, immunostaining, immunohistochemistry and cell fractionation

Western blotting was done by standard protocols as we have previously described [5, 10]. Relative band intensity was measured using Image J program. For immunostaining of endogenous CHTM1 protein, A549 cells were fixed, blocked with goat serum, and incubated with anti-CHTM1 antibody followed by FITC-labeled secondary antibody; the nuclei were counterstained with DAPI. To perform immunohistochemistry, paraffin embedded patient tissue slides were purchased from Biomax (Rockville, MD, USA) and staining was performed using Vector Vectastain kit following the manufacturer’s protocol as we have reported [5]. Quantification was performed by board certified pathologist. Mitochondrial and cytosolic fractionations were done as described previously [10].

### ROS and RNS level measurement

To measure oxidative stress, cells were stained with 1 μM DCF-DA (Invitrogen, CA, USA), a ROS sensitive dye or with 1 μM DAF-FM (Invitrogen, CA, USA) for 45 min at 37 °C followed by Hank’s balanced salt solution (HBSS) wash. Fluorescence intensity was measured with Ex/Em: 485/530 nm filter using Synergy 2 micro plate reader. In case of DCF-DA stained A549 lung cancer cells, live-cell confocal microscopy was performed using Zeiss LSM-780 microscope.

### Statistical analysis

All in vitro experiments are representative of at least 3 independent repeats. Values represent the mean ± SEM of three-independent experiments; for statistical significance 2-tailed Student’s t test or ANOVA was used. The *p* < 0.05 value was judged as statistically significant.

## Results

### CHTM1 deficiency increases lung cancer cell sensitivity to metabolic stress

**Fig. 1:**
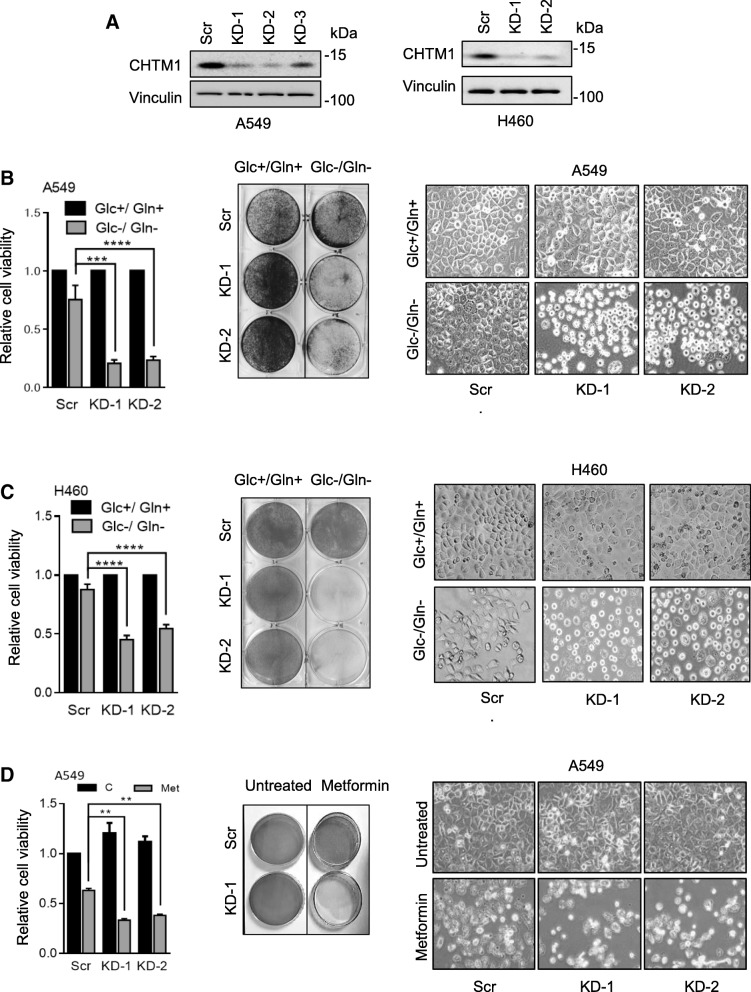
Alterations in CHTM1 levels affect sensitivity of lung cancer cells to glucose/glutamine starvation. (**a**) Western blot analyses showing CHTM1 knockdown in A549 and H460 lung cancer cells using three independent constructs. (**b&c**) CHTM1 knockdown and scrambled A549 and H460 lung cancer cells were glucose/glutamine starved for 6 h and 48 h respectively. Relative cell proliferation by MTT assay (*Left panels*), crystal violet staining (*Middle panels*) and representative phase-contrast photomicrographs (*Right panels*) showing decreased cell survival in CHTM1 knockdown cells compared to scramble cells under glucose/glutamine deprived condition. (**d**) CHTM1 knockdown and scramble A549 lung cancer cells were treated with 50 mM metformin for 48 h. *Left, middle* and *right panels* depict relative cell proliferation (MTT assay), crystal violet staining and representative phase-contrast photomicrographs respectively. CHTM1 knockdown cells show decreased cell survival following metformin treatment in comparison to metformin-treated scramble cells

We investigated the effect of CHTM1 deficiency on lung cancer cell sensitivity to metabolic stress using RNAi approach to first knockdown CHTM1 in A549 and H460 lung cancer cells. Western blots (Fig. 1a) show that CHTM1 was effectively knockdown in these cells. We cultured the CHTM1 knockdown and scrambled (control) cells in the absence of glucose/glutamine and noted that CHTM1 knockdown cells exhibited poor growth under glucose/glutamine deprivation as was noted by MTT assay (Fig. 1b&c*, left panel*), crystal violet staining (Fig. 1b&c, *middle panel*) and phase-contrast microscopy (Fig. 1b&c, *right panel*). We also investigated the role of CHTM1 in lung cancer cell response to a different metabolic stress inducer namely, metformin. Metformin is extensively used for the treatment of type 2 diabetes. Scramble (control) and CHTM1 knockdown A549 cells were treated with metformin and cell survival was analyzed. As is shown (Fig. 1d), CHTM1-deficient lung cancer cells exhibited poor growth also in response to metformin treatment. These results indicate that CHTM1 regulates cellular response to metabolic stress induced by metformin as well as glucose/glutamine deprivation.

### Metabolic stress-induced cell death in CHTM1-deficient cells is caspase-independent

**Fig. 2:**
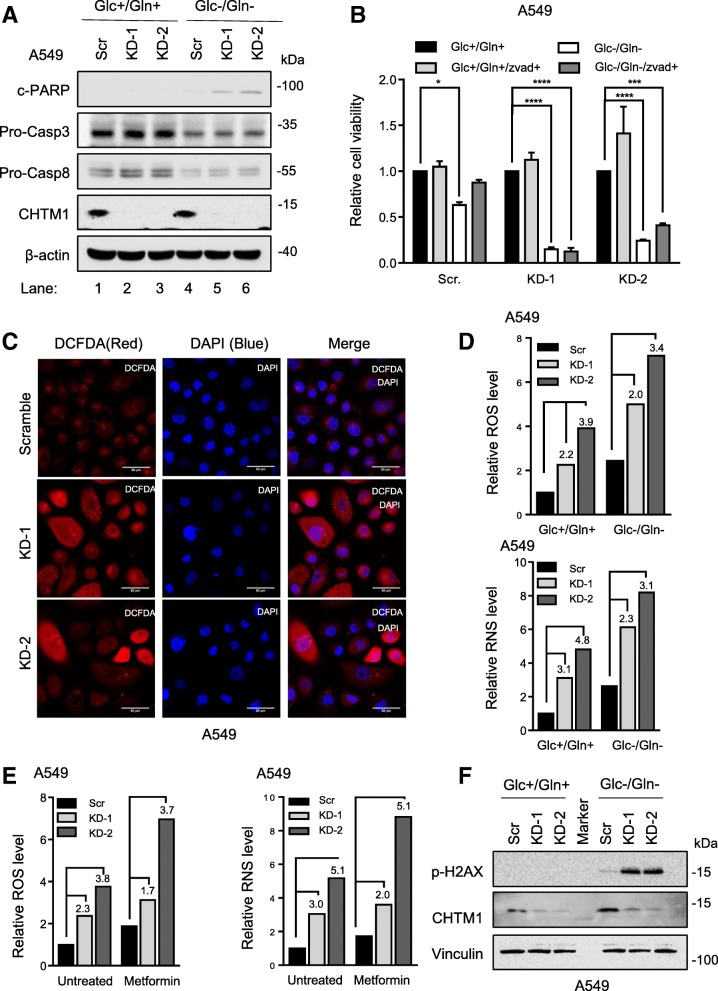
CHTM1 deficiency-associated metabolic stress-induced cell death is caspase-independent. CHTM1 knockdown and scrambled A549 lung cancer cells were growing in regular media or glucose/glutamine-depleted media (for 4 h). Western blot analyses (**a**) showing increase in PARP cleavage but no effect on procaspase levels in glucose/glutamine-starved CHTM1 knockdown cells. (**b**) MTT assay showing decreased cell survival of CHTM1 knockdown cells compared to scramble cells under glucose/glutamine-deprived conditions in the presence or absence of 20 μM Z-VAD-FMK (pan-caspase inhibitor). (**c**) Representative fluorescent photomicrographs showing increase in DCF-DA (red) stained reactive oxygen species in CHTM1 knockdown A549 cells. Scale bar, 50 μM (**d**) Relative levels of ROS and RNS in glucose/glutamine starved (for 4 h) CHTM1 knockdown A549 cells. (**e**) Relative levels of ROS and RNS in 50 mM metformin treated (12-h) CHTM1 knockdown A549 cells. DCF-DA for ROS and DAF-FM for RNS were used and analyses done by spectrophotometry. (**f**) Western blot analyses showing increased phosphorylation H2AX in CHTM1 knockdown cells under glucose/glutamine-deprived condition

Next, we investigated whether poor growth of CHTM1-deficient cells under metabolic stress was due to enhanced cell death involving activation of caspases. Our results (Fig. 2a), indicate that glucose/glutamine deprivation was associated with PARP cleavage, caspase 3 cleavage (Additional file 1: Figure S1A) and caspases 3 and 8 activation (decrease in procaspase levels) in scrambled cells (compare lanes 1&4). However, although PARP cleavage was further enhanced in CHTM1-deficient cells under glucose/glutamine deprivation (Fig. 2a top, compare lanes 4, 5, 6), caspases 3 and 8 activation did not further increase when compared to scrambled cells. We also investigated the effect of pan-caspase inhibitor Z-VAD-FMK on metabolic stress-induced growth inhibition in CHTM1-deficient and -proficient lung cancer cells. Our results (Fig. 2b) indicate that pretreatment with pan-caspase inhibitor Z-VAD-FMK effectively rescued from metabolic stress-induced growth inhibition in scrambled cells but only minimally affected CHTM1-deficient cells. CHTM1-deficient cells also exhibited down-regulation of cytochrome c and Smac levels under metabolic stress induced by glucose/glutamine deprivation (Additional file 1: Figure S1B) and metformin treatment (Additional file 1: Figure S1C). Taken together, these results suggest that metabolic stress-induced growth inhibition in CHTM1-deficient cells occurs due to cell death that does not appear to fully depend on caspase activation.

We also investigated whether metabolic stress-induced cell death was associated with increased oxidative stress. DCF-DA, a fluorogenic dye that measures the reactive oxygen species (ROS) was used. First, we stained the scramble and CHTM1 knockdown lung cancer cells growing in regular media with DCF-DA and noted that the CHTM1-deficient cells exhibited increased oxidative stress as was reflected by increased DCF-DA staining (Fig. 2c). The levels of ROS and reactive nitrogen species (RNS) in scramble and CHTM1 knockdown cells were also quantified following glucose/glutamine deprivation or metformin treatment. Results shown in Fig. 2d&e indicate that glucose/glutamine deprivation (Fig. 2d, *bar graphs in upper and lower panels*) or metformin treatment (Fig. 2e) increased ROS and RNS levels in both CHTM-proficient and CHTM1-deficient cells. However, CHTM1 depletion led to further elevation in ROS and RNS levels. Sustained high levels of ROS and RNS are known to cause cellular damage including DNA damage that can lead to cell death [11]. Accordingly, our results (Fig. 2f) also show that the levels of phospho-gamma-H2AX, a marker of DNA damage, significantly increased in the metabolically-stressed CHTM1-deficient cells. Thus, CHTM1 deficiency increases cell death under metabolic stress for which excessive oxidative stress also appears to play a role. Together, these findings highlight the important role for CHTM1 in promoting cell survival under metabolic stress in lung cancer cells.

### CHTM1 regulates cellular distribution of AIF1 in response to metabolic stress

**Fig. 3:**
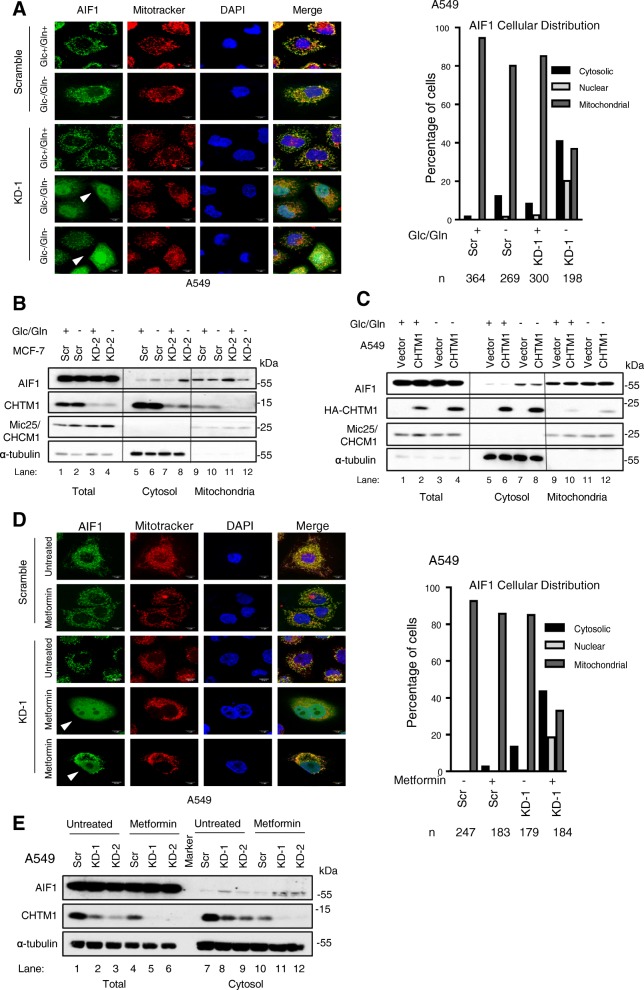
CHTM1 regulates AIF1 cellular distribution in response to metabolic stress**.** (**a**, *left panel*) Representative fluorescent photomicrographs showing increase in nucleo-cytosolic distribution of endogenous AIF1 (green) in CHTM1 knockdown A549 cells following glucose/glutamine starvation for 4 h. White arrows indicate cells with nuclear localization of AIF1. Scale bar, 10 μM. (*Right panel*) Quantitative results showing the relative numbers of A549 cells with cytosolic/nuclear/mitochondrial localization of AIF1 (n indicates the number of cells analyzed). (**b**) Representative Western blot showing increase in cytosolic AIF1 levels in CHTM1 knockdown MCF-7 breast cancer cells following 12-h glucose/glutamine starvation. (**c**) Representative Western blot showing decrease in levels of cytosolic AIF1 in CHTM1 overexpressing A549 lung cancer cells following 4-h glucose/glutamine starvation (compare lane 7 with 8). (**d**, *left panel*) Representative fluorescent photomicrographs showing increase in nucleo-cytosolic distribution of endogenous AIF1 (green) in CHTM1 knockdown A549 cells following 12-h metformin treatment. White arrows indicate cells with nuclear localization of AIF1. Scale bar, 10 μM. (*Right panel*) Quantitative results showing the relative numbers of A549 cells with cytosolic or nuclear localization of AIF1 following metformin treatment (n indicates the number of cells analyzed). (**e**) Representative Western blot analyses showing increase in AIF1 cytosolic levels in CHTM1 knockdown A549 cells upon 12-h metformin treatment (compare lane 10 with lanes 11&12)

AIF1 is a mitochondrial oxidoreductase that translocates from mitochondria to nucleus to induce caspase-independent cell death [12]. Our results indicate that metabolic stress-induced cell death in CHTM1-deficient cells appears to be caspase-independent (Fig. 2a&b). Next, we investigated the effect of CHTM1 deficiency on AIF1 subcellular distribution under metabolic stress using immunostaining approach. By immunostaining, mitochondrial AIF1 can be detected as exhibiting punctate distribution, whereas cytosolic AIF1 appears as diffuse. Our results (Fig. 3a) indicate that in A549 cells, AIF1 was mainly in association with mitochondria in both scramble and CHTM1 knockdown cells grown in complete media. However, cytosolic and nuclear distribution of AIF1 (Fig. 3a *left panel*, white arrows) was increased in CHTM1-knockdown cells in glucose/glutamine-deprived conditions when compared to scramble controls. To quantify these results, several hundred (~ 200–350) cells for each sample were counted and results (Fig. 3a *right panel*) indicated that the percentage of cells exhibiting AIF1 cytosolic/nuclear distribution was clearly increased in CHTM1-deficient cells under metabolic stress. Biochemical analyses were also performed to determine the subcellular distribution of AIF1; the results indicate that in MCF-7 breast cancer cells, AIF1 levels were increased in the cytosolic fractions of glucose/glutamine-deprived CHTM1-deficient cells (Fig. 3b, compare lanes 7&8) with concomitant decrease in the mitochondrial fractions (Fig. 3b, compare lanes 11&12). Cytosolic levels of AIF1 were also increased in glucose/glutamine-deprived CHTM1-deficient A549 cells (Additional file 1: Figure S2, compare lanes 3&4); interestingly, cytochrome c and Smac levels were reduced under these conditions (Additional file 1: Figure S2, compare lanes 3&4). Some cytochrome c and Smac were noted in the cytosolic fractions of unstressed cells (Additional file 1: Figure S2 lanes 1&2) as has also been reported in several other studies [13–15]. We also investigated the effect of exogenous CHTM1 on endogenous AIF1 levels in A549 cells and our results (Fig. 3c, compare lane 7&8) indicate that CHTM1 overexpression blunted the cytosolic accumulation of AIF1 under glucose/glutamine deprivation (of note, concentrations of samples representing cytosolic fractions in lanes 5–8 are compared to each other and not to mitochondrial or total fractions). Results of similar experiments using metformin indicated that metformin-induced metabolic stress also increased cytosolic and nuclear distribution of AIF1 in CHTM1 knockdown cells (Fig. 3d). Biochemical analyses performed on metformin-treated and untreated CHTM1-profieicnt and -deficient A549 cells also revealed increased cytosolic accumulation of AIF1 in CHTM1-deficient cells (Fig. 3e, lanes 10–12). Together, these findings suggest that CHTM1 appears to affect subcellular distribution of AIF1 under metabolic stress.

### CHTM1 interacts with AIF1

**Fig. 4:**
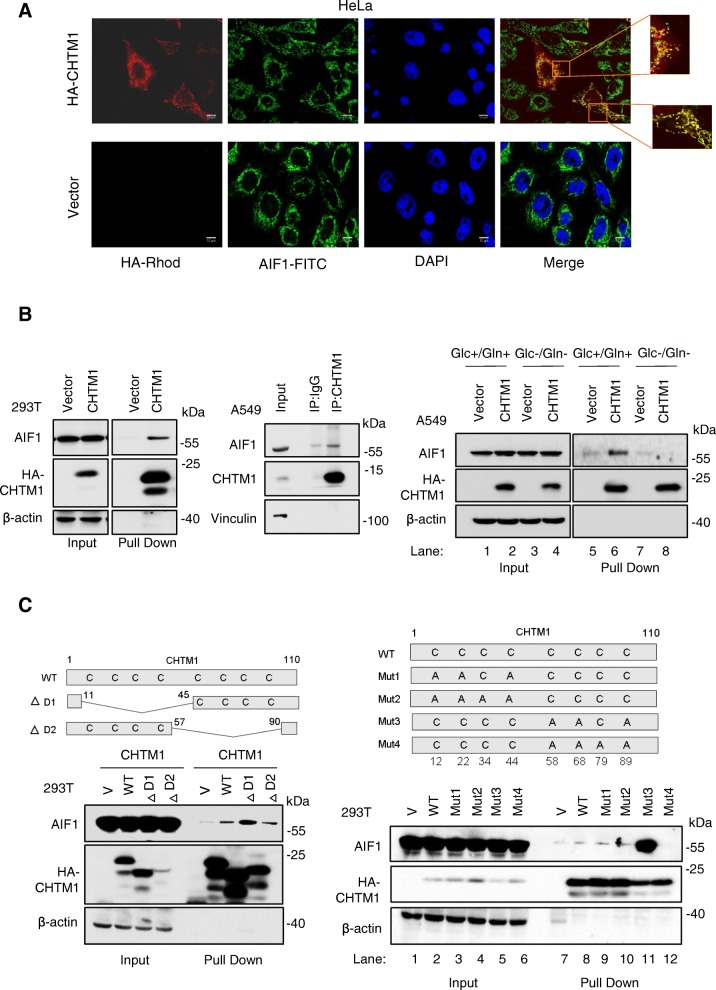
Metabolic stress regulates CHTM1-AIF1 interactions. (**a**) Representative fluorescent photomicrographs showing cellular localization of HA-tagged CHTM1 (Rhodamine stained, red) and endogenous AIF1 (FITC stained, green) in HeLa cells. Scale bar, 10 μM. (**b**, *left panel*) Western blot analyses of S-tag pull-down to demonstrate the interaction between endogenous AIF1 and exogenous HA-tagged CHTM1. (*Middle panel*) Western blot analyses of immunoprecipitation to demonstrate the interaction of endogenous AIF1 with endogenous CHTM1. (*Right panel*) Metabolic stress disrupts CHTM1-AIF1 interaction. S-tag pull-down assay was performed on A549 cells transiently transfected with HA-S-tagged CHTM1 or vector-only, and cultured in the presence or absence of glucose/glutamine for 4 h. (**c**, *left upper panel*) Schematic of deletion strategy in CHTM1 and Western blot analyses showing interactions between AIF1 and wild type (WT, full-length) or the deletion variants of CHTM1. (**c**, *left lower panel*) Western blot analyses showing interactions between AIF1 and wild type or the deletion variants of CHTM1. (**c**, *right upper panel*) Schematic of mutation strategy in CHTM1. (**c**, *right lower panel,* Western blot analyses showing increased AIF1 interaction with CHTM1 mutant, Mut3 harboring replacement of cysteine with alanine at positions 58, 68 and 89 (Lane 11). Decreased AIF1 interaction with CHTM1 mutant, Mut4 harboring replacement of cysteine with alanine at positions 58, 68, 79, 89 (Lane 12), indicating the contribution of residue C79 in CHTM1-AIF1 interaction

We also sought to investigate the potential mechanism by which CHTM1 affects subcellular distribution of AIF1 under metabolic stress. CHTM1 localizes in both cytosol and mitochondria although relative cytosolic and mitochondrial distribution varies from cell line to cell line as noted previously [5] and in the present study. Analysis of subcellular distributions of CHTM1 and AIF1 revealed that CHTM1 also co-localized with AIF1 in mitochondria (Fig. 4a). Therefore, we investigated whether CHTM1 interacted with AIF1. Biochemical analyses were performed using 293 T cells and exogenous CHTM1 co-precipitated with endogenous AIF1 (Fig. 4b, *left panel*). Using A549 cells, we also noted endogenous CHTM1 to co-immunoprecipitate with endogenous AIF1 (Fig. 4b, *middle panel*). These results therefore, demonstrate that CHTM1 and AIF1 interact with each other. Interestingly, we also noted that under glucose/glutamine-depleted condition (metabolic stress), CHTM1 and AIF1 interactions were reduced (Fig. 4b, *right panel;* compare lanes 5&6 with lanes 7&8).

We next examined the regions on CHTM1 responsible for its interactions with AIF1. CHTM1 harbors two CHCH domains (Fig. 4c, *left panel*); accordingly, we generated two CHTM1 deletion variants ΔD1 and ΔD2 lacking the CHCH domain 1 and CHCH domain 2 respectively (Fig. 4c, *left panel*). The deletion variants were used for assessing their interactions with endogenous AIF1. Figure 4c, *left panel* shows that deletion of either domain did not eliminate interaction with AIF1, a finding that suggests both CHCH domains to be involved in CHTM1 interaction with AIF1. We further investigated the molecular details of CHTM1-AIF1 interactions by introducing point mutations to replace critical cysteine residues with alanines (Cys➔Ala) in the CHCH domains of CHTM1 (Fig. 4c, *right panel*). The results (Fig. 4c, *right panel*) indicate that CHTM1 variant Mut3 harboring mutations (C58A, C68A and C89A) in the second CHCH domain exhibited increased binding to AIF1. Interestingly, we noted that one additional point mutation at residue C79 (Mut 4; C58A, C68A, C89A and C79A) abolished CHTM1 interactions with AIF1 (Fig. 4c, *right panel*). These results indicated the residue C79 to be critical in facilitating interactions between CHTM1 and AIF1.

### CHTM1 suppresses p38 activation and enhances cell survival during metabolic stress

**Fig. 5:**
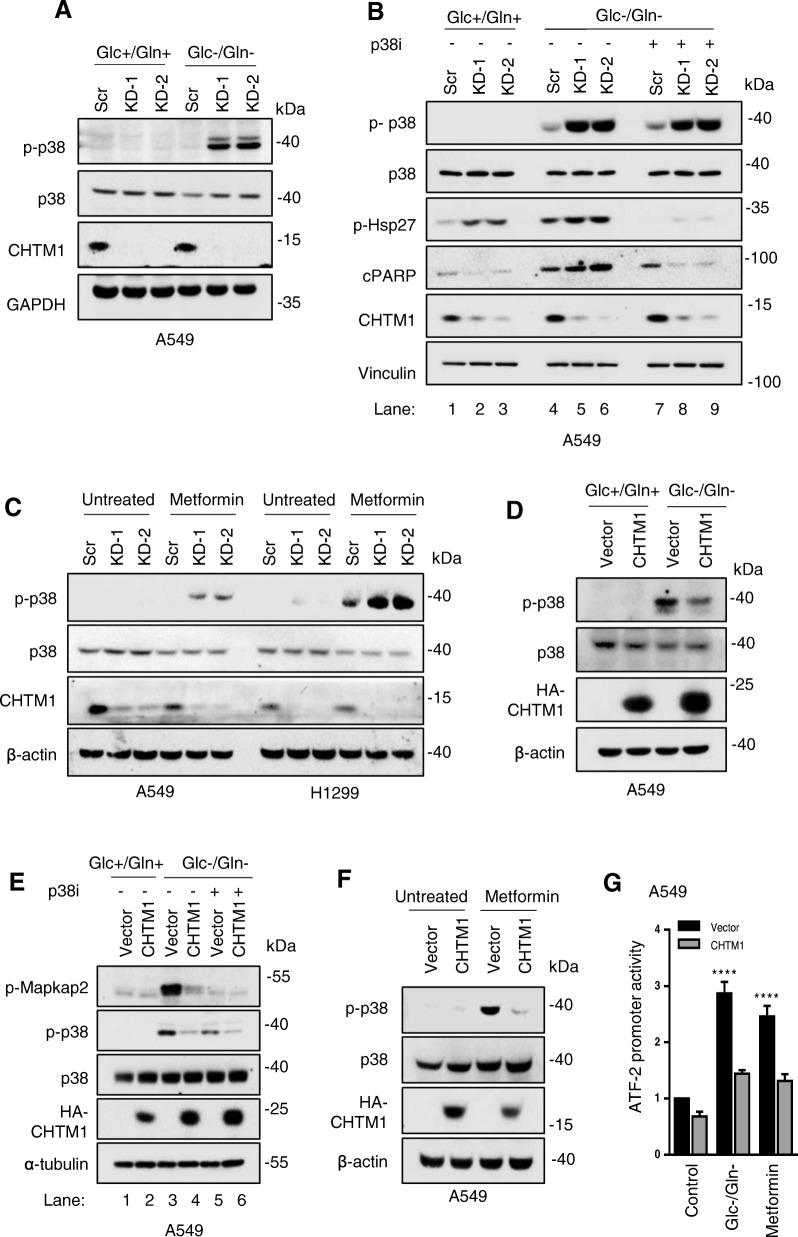
CHTM1 modulates p38 phosphorylation under metabolic stress condition. (**a**) Western blot analyses showing increased p38 phosphorylation in CHTM1 knockdown A549 cells following 4-h glucose/glutamine starvation (**b**) Western blot analyses showing p38 inhibitor SB203580 (p38i) abrogates Hsp27 phosphorylation in CHTM1 knockdown A549 cells (lanes 7–9). Cells were pretreated with the inhibitor (10 μM) for 2 h and then starved for glucose/glutamine for 4 h. (**c**) Western blot analyses showing increased p38 phosphorylation in CHTM1 knockdown A549 and H1299 lung cancer cells following 50 mM metformin treatment for 12 h. (**d**) Western blot analyses showing decrease in p38 phosphorylation in CHTM1 overexpressing A549 cells starved for glucose/glutamine for 4 h. (**e**) Western blot analyses showing p38 inhibitor SB203580 (p38i) abrogates MAPKAP2 phosphorylation in CHTM1 overexpressing A549 cells (compare lanes 3&4). Cells were pretreated with the inhibitor (10 μM) for 2 h and then starved for glucose/glutamine for 4 h. (**f**) Western blot analyses showing decreased p38 phosphorylation in CHTM1 overexpressing A549 cells following 50 mM metformin treatment for 12 h. (**g**) CHTM1 overexpression regulates p38 activity under metabolic stress. A549 cells were co-transfected with CHTM1 or empty vector and ATF2 promoter luciferase construct for 48 h and luciferase assay was performed 4-h after glucose/glutamine deprivation or 12-h after 50 mM metformin

p38 kinase is activated by various stresses including glucose starvation and metformin treatment [16, 17], and its activation is crucial for cell death following certain cellular stresses [18, 19]. To gain further mechanistic insights into the how CHTM1 alters cell survival following metabolic stress, we investigated the possible link between CHTM1 and p38 activation. Figure 5a shows that p38 phosphorylation was strongly induced in CHTM1-deficient cells cultured in glucose/glutamine-deprived medium (under metabolic stress) compared to those in regular growth medium. CHTM1 deficiency also led to enhanced phosphorylation of Hsp27 (a p38 substrate) and increased PARP cleavage (cPARP) under glucose/glutamine deprivation (Fig. 5b, lanes 4, 5 and 6) and these effects were reversed by p38 inhibitor SB203580 (Fig. 5b, compare lanes 4, 5 and 6 with lanes 7, 8 and 9). In reverse experiments, overexpression of CHTM1 suppressed glucose/glutamine withdrawal-mediated p38 activation (Fig. 5c) as well as phosphorylation on MAPKAP2 (a p38 substrate) (Fig. 5d, lanes 3&4). CHTM1 deficiency also enhanced p38 phosphorylation in metformin-treated lung cancer cells (Fig. 5e), and overexpression of CHTM1 inhibited p38 phosphorylation in metformin-treated cells (Fig. 5f). These results indicate that CHTM1 is a negative regulator of p38. ATF2 is a downstream target of p38; we also utilized luciferase reporting system (see Methods) to analyze the activation of ATF2 and thus, p38 activation. Our results (Fig. 5g) indicate that both glucose/glutamine starvation and metformin treatment induced ATF2 activation that was inhibited by CHTM1 overexpression. Together these results indicate that suppression of p38 activation appears to be an important mechanism via which CHTM1 promotes cell survival under metabolic stress. Consistent with this notion, activated p38 has been reported to alter the transcription of pro-apoptotic genes to modulate stress-induced cell death [20].

### CHTM1 modulates p38 activity and AIF1 subcellular distribution to promote cell survival under metabolic stress

**Fig. 6:**
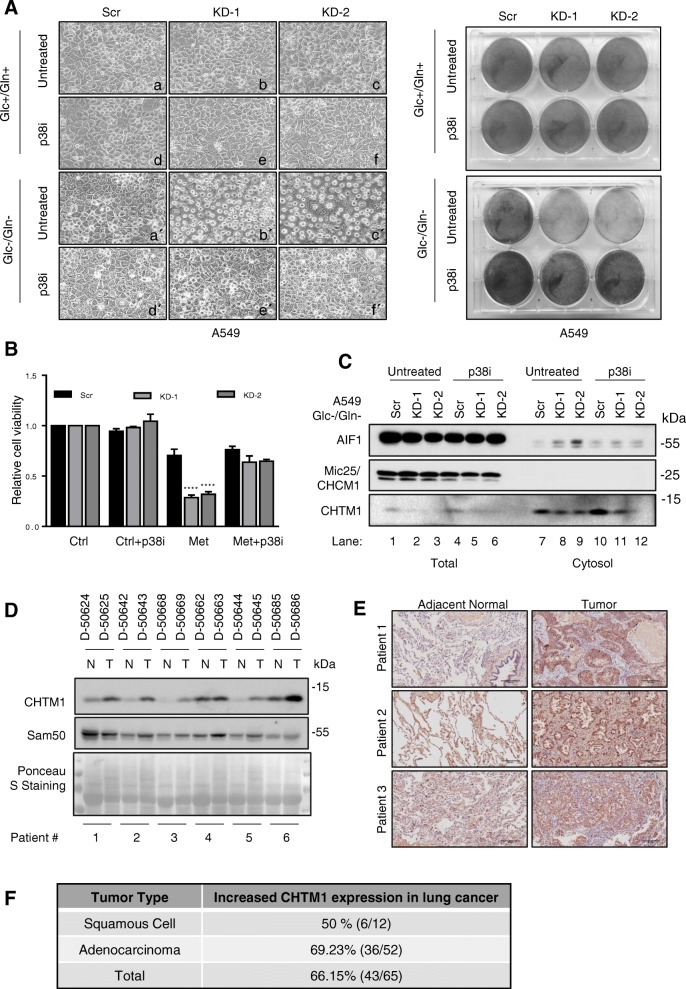
CHTM1 involves p38-AIF1 to modulate metabolic stress-induced cell death and is deregulated in human lung cancers. CHTM1-knockdown and scrambled A549 cells were glucose/glutamine starved for 4 h in the presence and absence of p38 kinase inhibitor SB203580. (**a**, *left panel*) Representative phase contrast photomicrographs, and (*Right panel*) crystal violet staining showing glucose/glutamine starvation-induced cell death was abrogated by p38 inhibitor SB203580 in CHTM1-deficient A549 cells. (**b**) Trypan blue exclusion assay showing metformin-induced cell death was prevented by p38 inhibitor SB203580 in CHTM1-deficient A549 cells. (**c**) Western blot analyses showing p38 inhibition blocks glucose/glutamine starvation-induced cytosolic accumulation of AIF1 in CHTM1 knockdown A549 cells (compare lanes 8&9 with lanes 11&12). (**d**) Representative Western blot showing CHTM1 expression in matching tumor (T) and adjacent normal (N) from same lung cancer patients. Same blot was also probed with anti-Sam50, another mitochondrial protein. As is shown, Sam50 does not show expression pattern similar to CHTM1, suggesting that the increase in CHTM1 is not due to generalized increase in mitochondrial contents. Samples were obtained from the Cooperative Human Tissue Network, an NCI supported network. (**e**) Immunohistochemistry-based detection of CHTM1 (brown color) in representative normal and tumor tissues from lung cancer patients. Samples were also stained with hematoxylin (blue color). Scale bar, 50 μM. Samples were purchased from Biomax (Rockville, MD) as formalin-fixed, paraffin-embedded tissue array slides. (**f**) Overall results of CHTM1 overexpression in lung cancer samples compared to matched normal tissue samples

We also investigated the interplay between CHTM1 and p38 in relation to metabolic stress-induced cell death. In this context, our results indicate that in CHTM1-deficient A549 cells, the metabolic stress-induced cell death was prevented by p38 inhibitor SB203580 (Fig. 6a *left panel*, compare panels b’&c’ with e’&f’; see also *right panel*). SB203580 also substantially increased survival of metformin-treated CHTM1-deficicent cells (Fig. 6b). Together these results suggest that CHTM1 deficiency was associated with increased cell death under metabolic stress that appeared to occur due to stronger activation of p38, and p38 inhibition blocked the death-inducing effects of p38. Our preceding results (Fig. 3) indicated that cytosolic and nuclear accumulation of AIF1 was increased in CHTM1-deficient cells under metabolic stress. Here, we noted that while p38 inhibitor did not alter total cellular levels of AIF1 in CHTM1-deficient cells under metabolic stress, it inhibited cytosolic accumulation of AIF1 (Fig. 6c, compare lanes 8&9 with lanes 11&12). Thus, p38 inhibition resulted in decreased accumulation of cytosolic AIF1 in metabolically-stressed CHTM1-deficient cells. These findings suggest that in CHTM1-deficient lung cancer cells, p38 activation appears to play a critical role in regulation of subcellular distribution of AIF1 under metabolic stress. These findings also suggest that CHTM1 regulates p38 activity as well as AIF1 subcellular distribution to mediate cell survival under metabolic stress.

### CHTM1 is deregulated in human lung cancer

We also investigated the expression status of CHTM1 in lung cancer patient samples. CHTM1 levels in lung cancer patient samples were analyzed by Western blotting and immunohistochemically staining. Figure 6d shows a representative Western blot with samples from 6 lung cancer patients in pairs as tumor and matching normal tissues. As is shown, tumor samples from 5 patients showed increased CHTM1 levels compared to their matching normal tissues (Fig. 6d). Overall, we analyzed matched normal and tumor tissues from 29 patients via Western blotting and found 24/29 (82.75%) patients had increased CHTM1 levels in their tumors. We also analyzed the CHTM1 status in patient samples by immunohistochemical staining. First, the specificity of the anti-CHTM1 antibody was confirmed via immunohistochemical staining performed on scrambled and CHTM1 knockdown A549 cells (Additional file 1: Figure S3A). The anti-CHTM1 antibody detected the immunohistochemistry-based signals in the scrambled cells but not in CHTM1 knockdown cells (Additional file 1: Figure S3A). Furthermore, only the anti-CHTM1 antibody detected the CHTM1-specific endogenous signals, whereas isotype-matched IgG did not (Additional file 1: Figure S3A). After confirming the specificity of the anti-CHTM1 antibody, we analyzed 36 lung cancer patient samples by immunohistochemical staining; our results indicated that 18/36 (50%) tumor specimens exhibited increased CHTM1 level. Figure 6e shows representative photomicrographs of immunohistochemical staining on tumors and adjacent normal specimens from 3 lung cancer patients. Our overall results based on Western blotting and immunohistochemistry are in Fig. 6f and also in supplementary information (Additional file 1: Figure S3B, Additional file 1: Table S1 and S2). Collectively, 43/65 (66.15%) lung cancer patient specimens had increased CHTM1 levels indicating that CHTM1 expression is elevated in the majority of human lung cancer samples analyzed.

## Discussion

In this manuscript, we report that CHTM1 is a novel modulator of metabolic stress as its deficiency sensitized human lung cancer cells to metabolic stress-induced cell death mediated by glucose/glutamine deprivation and metformin treatment (Fig. 1). In CHTM1-proficient cells, metabolic stress-induced cell death was coupled with caspases 3 and 8 activation and PARP cleavage but in CHTM1-deficient cells these caspases were not further activated although PARP cleavage was increased (Fig. 2a). Pan-caspase inhibitor also did not effectively rescue CHTM1-deficient cells from metabolic stress-mediated cell death (Fig. 2b). Thus, in CHTM1-deficient cells metabolic stress-induced cell death appears to occur in a caspase activation-independent manner. Our present study has identified a novel mechanism via which CHTM1 modulates cell death triggered by metabolic stress. We have shown that CHTM1 and AIF1 interact with each other (Fig. 4). Under metabolic stress, CHTM1-deficient condition results in increased cytosolic and nuclear accumulation of AIF1 (Fig. 3&6c), unlike cytochrome c and Smac (Additional file 1: Figure S2), indicating that CHTM1 modulates the subcellular distribution of AIF1. Of note, phospho-gamma-H2AX levels are also increased in CHTM1-deficient cells under these conditions (Fig. 2f).

AIF1 is an important death inducing molecule residing in the mitochondria under unstressed condition [21]. Following death stimuli, AIF1 is released from mitochondria into cytosol and then translocates to nucleus to mediate chromatin condensation and DNA fragmentation, suggested by increase in phospho-gamma-H2AX levels, and thus, caspase-independent cell death [22]. Our results suggest that metabolic stress-induced cell death in CHTM1-deficient cancer cells is predominantly associated with AIF1 modulation and not cytochrome c or Smac alterations. AIF1 has been shown to interact with CHCHD4, another protein from the CHTM1 family, to modulate CHCHD4 mitochondrial import and activity [23]. Our results suggest that CHTM1, as an important modulator of metabolic stress response, interacts with AIF1 and thus could keep AIF1 in association with mitochondria under metabolic stress. Accordingly, CHTM1 appears to negatively regulate AIF1 by preventing AIF1 translocation to the cytosol/nucleus and inhibiting AIF1-mediated caspase-independent cell death. In this context, we note that in CHTM1-proficient cells, the interactions between CHTM1 and AIF1 are blunted under metabolic stress but not fully abolished (Fig. 4b, *right panel*). It is therefore, possible that in CHTM1-proficient cells, some AIF1 is still able to translocate to cytosol/nucleus to mount apoptotic effects under metabolic stress. In CHTM1-deficient cells, however, due to CHTM1 absence, the AIF1 translocation from mitochondria towards cytosol/nucleus would be more efficient and could explain increased cell death in CHTM1-deficient cells under metabolic stress.

Our results also indicate that under metabolic stress, CHTM1 appears to modulate p38 activation to control AIF1 subcellular distribution. We show that CHTM1 deficiency results in increased phosphorylation of p38 and p38 substrate Hsp27 (Fig. 5a&b). The p38 inhibitor inhibits CHTM1 deficiency-induced Hsp27 phosphorylation, PARP cleavage (Fig. 5b) and cytosolic accumulation of AIF1 (Fig. 6c). Overexpression of CHTM1 also inhibits metabolic stress-induced phosphorylation of p38 and p38 substrate pMapkap2 (Fig. 5d-f), and ATF2 transactivation mediated by p38 induction (Fig. 5g). Importantly, p38 inhibition also overrides the influence of CHTM1 deficiency-mediated p38 activation and rescues the CHTM1-deficient lung cancer cells from metabolic stress-induced cell death (Fig. 6a&b). Taken together, these findings indicate that p38 activation plays an important role in metabolic stress-induced AIF1 subcellular distribution and cell death. Based on our results, a model is proposed that under metabolic stress, CHTM1 promotes cell survival by regulating p38 activity and attenuating the release of AIF1 from mitochondria (Additional file 1: Figure S4).

Our findings in this study highlight a novel CHTM1-mediated regulatory pathway via which CHTM1 overexpressing lung cancer cells could escape cell death under nutrition-deficient condition. In this context, it is of note that we also found CHTM1 expression to be significantly elevated in the majority (66.15%, 43/65) of lung cancers compared to their matching normal tissues. Out of 65 pairs of normal lung and lung cancer tissues analyzed, CHTM1 overexpression was noted in 6/12 (50%) squamous cell carcinomas and 36/52 (69.2%) adenocarcinomas (Fig. 6f); one large cell carcinoma sample also exhibited elevated CHTM1 levels. Although these results suggest that CHTM1 is deregulated in both squamous cell carcinomas and adenocarcinomas, further studies using a larger cohort of patient samples will provide further insight into the relative expression status of CHTM1 in these histological types.

The finding that CHTM1 is deregulated in lung cancer is clinically relevant because CHTM1 is a novel modulator of metabolic stress response and a metabolic marker. One could envision scenarios for established tumors with limited blood supply and/or for newly metastasized tumor cells at secondary sites without neoangiogenesis. In such situations, increased CHTM1 levels are expected to provide growth/survival advantage in nutrient-deficient environment to promote tumor growth. Given that reduction of CHTM1 levels leads to poor survival of metabolically-stressed lung cancer cells as reported here, pharmacologic or genetic targeting of CHTM1 could be a viable approach to manage this malignancy.

Our results also indicate that CHTM1 deficiency sensitizes human lung cancer cells to metformin. Metformin, a safe drug, is used for type 2 diabetes. Accordingly, World Health Organization lists metformin as one of the essential medicines. Metformin has also shown anticancer potential and there is interest to repurpose it for the treatment and prevention of human cancers. Regarding metformin for treatment of human malignancy, several clinical trials are ongoing including also for lung cancer (ClinicalTrials.gov). Clearly, metformin also shows anticancer potential, however, the molecular mechanisms of its anticancer effects remain to be fully elucidated. In this context, our results indicate that CHTM1 is an important modulator of metabolic stress response that is also capable of altering lung cancer cell sensitivity to metformin. For example, CHTM1-deficient lung cancer cells became more sensitive to the growth inhibitory effects of metformin. Given that metformin induces metabolic stress, it is tempting to propose that the sensitivity of lung cancer cells to drugs such as metformin can be enhanced if CHTM1 is genetically or pharmacologically antagonized. Clearly, CHTM1 could be considered as a valuable target to test novel anticancer therapeutics and improve the use of existing ones.

## Conclusion

Thus, CHTM1 appears to be an important metabolic marker that regulates cancer cell survival under metabolic stress conditions, and has the potential to be developed as a predictive tumor marker.

## Additional file

Additional file 1: **Figure S1.** Effect of CHTM1 deficiency on cleaved caspase 3, total Smac, total cytochrome c levels in A549 cells. **Figure S2.** Cytosolic levels of AIF1 are increased in glucose/glutamine deprived CHTM1 knockdown A549 cells. **Figure S3.** CHTM1 levels are upregulated in lung cancer. **Figure S4.** Schematic of hypothetical model showing the role of CHTM1 in modulating cancer death under metabolic stress. **Table S1.** Clinicopathological features of matching normal and tumor tissues from lung cancer patients evaluated by western blot analysis. **Table S2.** Clinicopathological features of matching normal and tumor tissues from lung cancer patients evaluated by immunohistochemistry. (PDF 947 kb)

## Data Availability

All data generated during this study are included in this published article and in supplementary information files.

## Abbreviations

AIF1: Apoptosis Inducing Factor 1 (AIF1)
CHCM1: Coiled-coil Helix Cristae Morphology 1
CHTM1: Coiled-coil Helix Tumor and Metabolism 1
cPARP: Cleaved PARP
Glc/Gln: Glucose/glutamine
KD: Knockdown
MAPKAP2: Mitogen-Activated Protein Kinase-Activated Protein Kinase 2
OXPHOS: Oxidative phosphorylation
RNS: Reactive Nitrogen Species
ROS: Reactive oxygen species
Scr: Scramble
