# Supplementary material for: CHTM1 regulates cancer cell sensitivity to metabolic stress via p38-AIF1 pathway
Source: J Exp Clin Cancer Res. 2019 Jun 20;38:271. doi: 10.1186/s13046-019-1253-5 (PMC6587271; doi:10.1186/s13046-019-1253-5)
Supplement: Supplementary file 1 — Figure S1. Effect of CHTM1 deficiency on cleaved caspase 3, total Smac, total cytochrome c levels in A549 cells. Figure S2. Cytosolic levels of AIF1 are increased in glucose/glutamine deprived CHTM1 knockdown A549 cells. Figure S3. CHTM1 levels are upregulated in lung cancer. Figure S4. Schematic of hypothetical model showing the role of CHTM1 in modulating cancer death under metabolic stress. Table S1. Clinicopathological features of matching normal and tumor tissues from lung cancer patients evaluated by western blot analysis. Table S2. Clinicopathological features of matching normal and tumor tissues from lung cancer patients evaluated by immunohistochemistry. (PDF 947 kb) [file 13046_2019_1253_MOESM1_ESM.pdf]

Figure S1

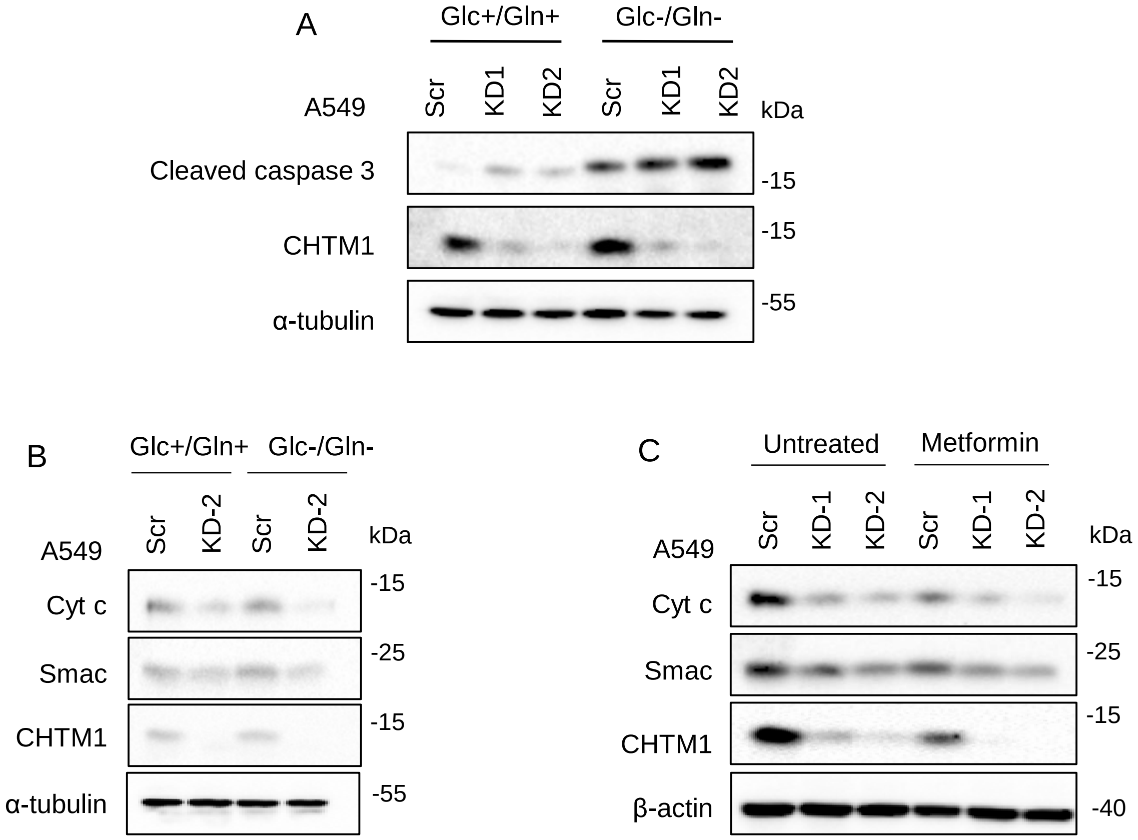

Figure S1. Effect of CHTM1 deficiency on cleaved caspase 3, total Smac and total cytochrome c levels in A549 cells. (A) Representative Western blots showing caspase 3 cleavage in CHTM1 knockdown (KD) A549 cells grown in regular media or following 4 hours of glucose/glutamine starvation. (B) Representative Western blots showing decrease in total cytochrome c and Smac levels in CHTM1 knockdown (KD) A549 cells grown in regular media or following 4 hours of glucose/glutamine starvation. (C) Representative Western blot showing decrease in cytochrome c and Smac levels in CHTM1 knockdown A549 cells following 12 hours of metformin treatment.

Figure S2

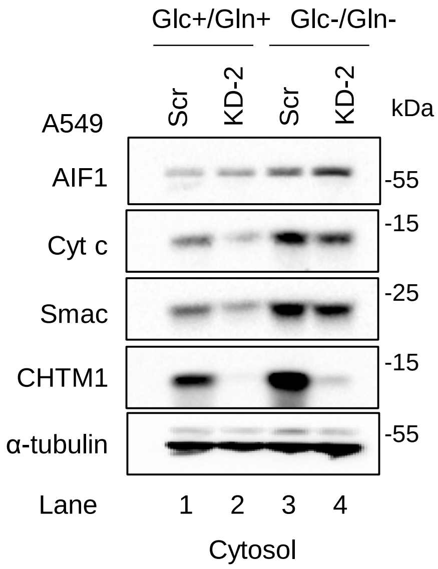

Figure S2. Cytosolic levels of AIF1 are increased in glucose/glutamine-deprived CHTM1 knockdown A549 cells. Representative Western blots showing increase in AIF1 levels, and decrease in cytochrome c and Smac levels in cytosolic fractions of CHTM1 knockdown (KD) A549 cells following 4 hours of glucose/glutamine starvation (lanes 3 and 4). Some cytochrome c and Smac were noted in the cytosolic fractions of unstressed cells (lanes 1 and 2) as has also been reported in several other studies (refs.11-13).

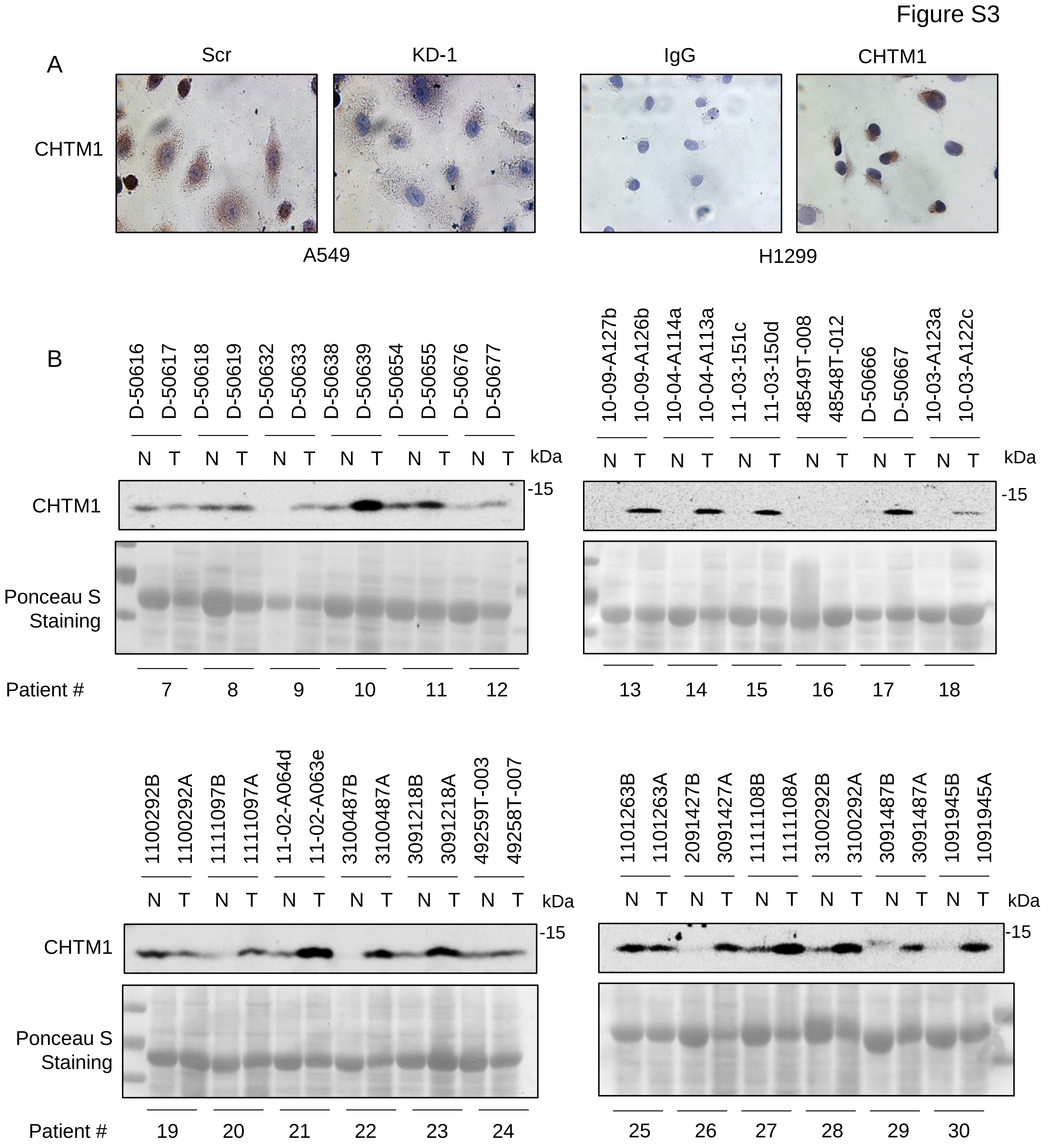

Figure S3. CHTM1 levels are upregulated in lung cancer. (A) Left panel; Immunohistochemical staining of scramble and CHTM1 knockdown A549 cells as described in Material and Methods. Right panel: Immunohistochemical staining of H1299 cells stained with isotype-matched IgG and anti-CHTM1 antibodies as describe in Material and Methods. (B) Western blots showing CHTM1 expression in matched normal (N) and tumor (T) tissues from lung cancer patients (Patient #7-30).

Figure S4

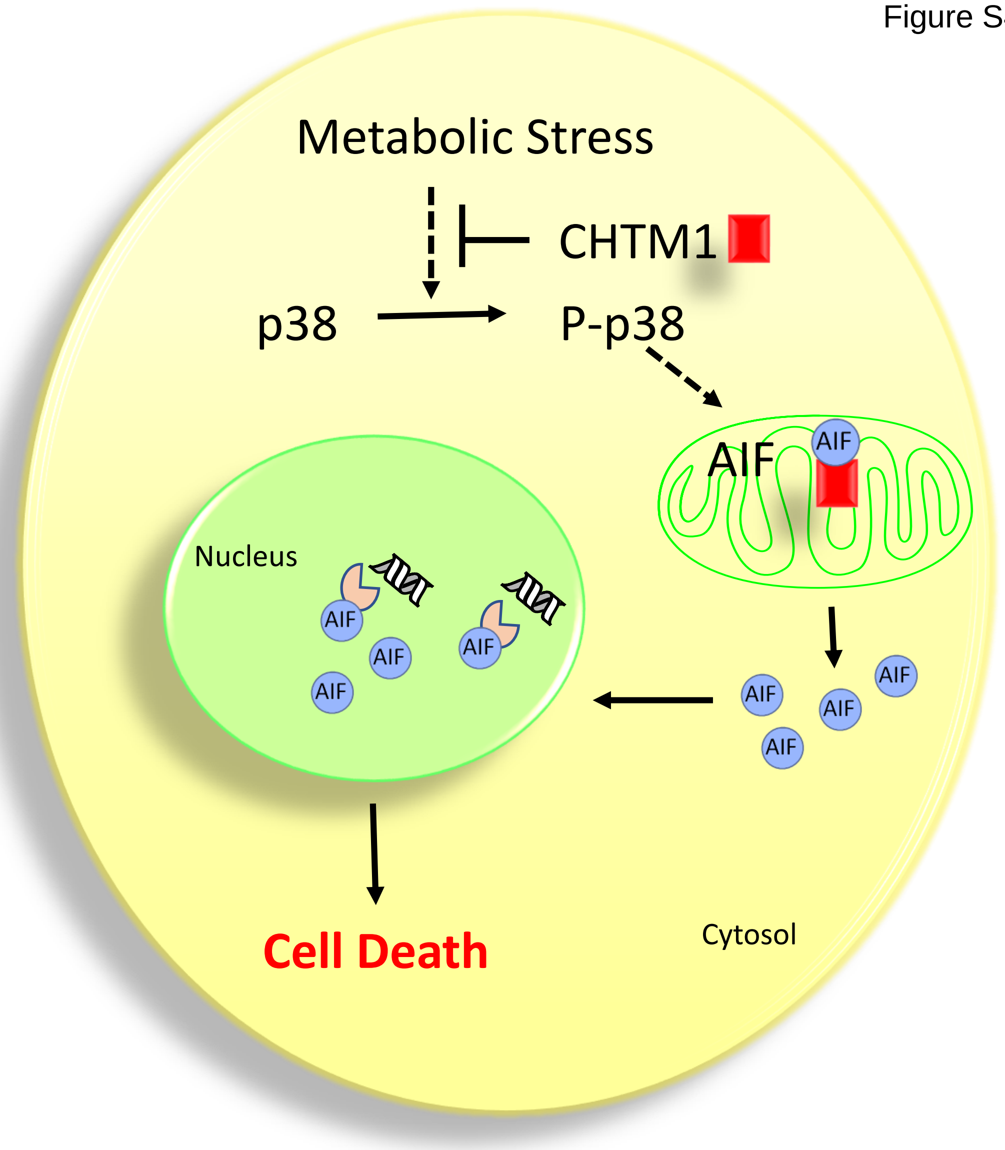

Figure S4. Schematic of hypothetical model showing the role of CHTM1 in modulating cancer death under metabolic stress. As is shown, CHTM1 inhibits metabolic stress-induced p38 phosphorylation/activation. CHTM1 also interacts with AIF1 and prevents AIF1 release out of mitochondria under metabolic stress. CHTM1 deficiency enhances AIF1 cytosolic and nuclear accumulation and thereby induces caspase-independent cell death under metabolic stress in lung cancer cells.

Table S1. Clinicopathological features of matching normal and tumor tissues from lung cancer patients evaluated by Western blot analyses

Patient #	Patient ID	Type	Age	Sex	CHTM1 Status in tumor
1	D-50624	Normal	72	Male	Increase
	D-50625	Squamous Cell Carcinoma	72	Male	
2	D-50642	Normal	76	Male	Increase
	D-50643	Large cell carcinoma	76	Male	
3	D-50668	Normal	48	Male	Increase
	D-50669	Adenocarcinoma	48	Male	
4	D-50662	Normal	70	Male	No Change
	D-50663	Adenocarcinoma	70	Male	
5	D-50644	Normal	70	Male	Increase
	D-50645	Squamous Cell Carcinoma	70	Male	
6	D-50685	Normal	68	Female	Increase
	D-50686	Adenocarcinoma	68	Female	
7	D-50616	Normal	68	Male	Decrease
	D-50617	Squamous Cell Carcinoma	68	Male	
8	D-50618	Normal	76	Male	No Change
	D-50619	Squamous Cell Carcinoma	76	Male	
9	D-50632	Normal	80	Female	Increase
	D-50633	Squamous Cell Carcinoma	80	Female	
10	D-50638	Normal	66	Male	Increase
	D-50639	Squamous Cell Carcinoma	66	Male	
11	D-50654	Normal	60	Male	Increase
	D-50655	Adenocarcinoma	60	Male	
12	D-50676	Normal	78	Male	Increase
	D-50677	Squamous Cell Carcinoma	78	Male	
13	10-09-A127b l	Normal	74	Female	Increase
	10-09-A126b	Adenocarcinoma	74	Female	
14	10-04-A114a	Normal	68	Male	Increase
	10-04-A113a	Adenocarcinoma	68	Male	
15	11-03-151c	Normal	66	Female	Increase

	11-03-150d	Adenocarcinoma	66	Female	
17	D-50666	Normal	47	Male	Increase
	D-50667	Adenocarcinoma	47	Male	
18	10-03-A123a	Normal	68	Male	Increase
	10-03-A122c	Adenocarcinoma	68	Male	
19	1100292B	Normal	67	Male	Decrease
	1100292A	Adenocarcinoma	67	Male	
20	1111097B	Normal	74	Male	Increase
	1111097A	Adenocarcinoma	74	Male	
21	11-02-A064d	Normal	67	Male	Increase
	11-02-A063e	Adenocarcinoma	67	Male	
22	3100487B	Normal	73	Female	Increase
	3100487A	Adenocarcinoma	73	Female	
23	3091218B	Normal	66	Female	Increase
	3091218A	Adenocarcinoma	66	Female	
24	49259T-003	Normal	60	Female	Increase
	49258T-007	Adenocarcinoma	60	Female	
25	1101263B	Normal	75	Female	No Change
	1101263A	Adenocarcinoma	75	Female	
26	2091427B	Normal	57	Female	Increase
	3091427A	Adenocarcinoma	57	Female	
27	1111108B	Normal	76	Male	Increase
	1111108A	Adenocarcinoma	76	Male	
28	3100292B	Normal	54	Male	Increase
	3100292A	Adenocarcinoma	54	Male	
29	3091487B	Normal	68	Male	Increase
	3091487A	Adenocarcinoma	68	Male	
30	1091945B	Normal	75	Female	Increase
	1091945A	Adenocarcinoma	75	Female	

Table S2. Clinicopathological features of matching normal and tumor tissues from lung cancer patients evaluated by immunohistochemistry. Tissue array slides were supplied by Biomax. CHTM1 was detected by anti-CHTM1 antibody. The results were evaluated by a board-certified pathologist.

Patient #	Pathology diagnosis	Age	Sex	TNM	Grade	Stage	CHTM1 Status
BM-1	Squamous cell carcinoma	64	M	T3N1M1	3	IV	No Change
	Normal Adjacent						
BM-2	Squamous cell carcinoma	66	M	T2N0M0	2	I	No Change
	Pulmonary edema with congestion						
BM-3	Squamous cell carcinoma	67	M	T3N0M0	3	IIB	No Change
	Normal Adjacent						
BM-4	Squamous cell carcinoma	49	M	T2N1M0	3	IIB	No Change
	Normal Adjacent						
BM-5	Squamous cell carcinoma	53	M	T2N0M0	3	IB	No Change
	Normal Adjacent						
BM-6	Adenocarcinoma	42	M	T2N0M0	3	IB	No Change
	Normal Adjacent						
BM-7	Adenocarcinoma	66	F	T1N0M0	G1	IA	Increased
	Normal Adjacent						
BM-8	Adenocarcinoma	68	F	T1N0M0	G1	IA	Increased
	Normal Adjacent						
BM-9	Adenocarcinoma	69	F	T1N0M0	G1	IA	Increased
	Normal Adjacent						
BM-10	Adenocarcinoma	58	F	T3N0M0	G1-G2	IIB	Increased
	Normal Adjacent						
BM-11	Adenocarcinoma	63	F	T1NxM0	G1-G2	II-III	Increased
	Normal Adjacent						
BM-12	Adenocarcinoma	71	F	T1NxM0	G2	II-III	No Change
	Normal Adjacent						
BM-13	Adenocarcinoma	51	M	T1NxM0	G2	II-III	Increased
	Normal Adjacent						
BM-14	Adenocarcinoma	56	F	T2aNxM0	G2	II-III	Increased
	Normal Adjacent						
BM-15	Adenocarcinoma	30	F	T2aNxM0	G2	II-III	Increased

	Normal Adjacent						
BM-16	Adenocarcinoma<br>Normal Adjacent	42	M	T1NxM0	G2	II-III	Increased
BM-17	Adenocarcinoma<br>Normal Adjacent	63	M	T1NxM0	G2	II-III	Increased
BM-18	Adenocarcinoma<br>Normal Adjacent	57	F	T2aNxM0	G2	II-III	Increased
BM-19	Adenocarcinoma<br>Normal Adjacent	51	F	T2aNxM0	G2	II-III	Increased
BM-20	Adenocarcinoma<br>Normal Adjacent	60	F	T1NxM0	G2	II-III	No Change
BM-21	Adenocarcinoma<br>Normal Adjacent	61	F	T1NxM0	G2	II-III	No Change
BM-22	Adenocarcinoma<br>Normal Adjacent	50	F	T1NxM0	G2	II-III	Increased
BM-23	Adenocarcinoma<br>Normal Adjacent	70	M	T2aNxM0	G2	II-III	No Change
BM-24	Adenocarcinoma<br>Normal Adjacent	59	M	T2aNxM0	G2	II-III	Increased
BM-25	Adenocarcinoma<br>Normal Adjacent	40	M	T1NxM0	G2	II-III	No Change
BM-26	Adenocarcinoma<br>Normal Adjacent	62	F	T1NxM0	G2	II-III	No Change
BM-27	Adenocarcinoma<br>Normal Adjacent	52	F	T2aNxM0	G2-G3	II-III	Increased
BM-28	Adenocarcinoma<br>Normal Adjacent	63	F	T2aNxM0	G2-G3	II-III	No Change
BM-29	Adenocarcinoma<br>Normal Adjacent	60	M	T1NxM0	G2-G3	II-III	No Change
BM-30	Adenocarcinoma<br>Normal Adjacent	60	F	T1M0	G2-G3		No Change
BM-31	Adenocarcinoma<br>Normal Adjacent	37	F	T2aM0	G3		Increased
BM-32	Adenocarcinoma<br>Normal Adjacent	67	M	T2bNxM0	G3	II-III	Increased
BM-33	Adenocarcinoma<br>Normal Adjacent	63	F	T2aNxM0	G3	II-III	No Change
BM-34	Adenocarcinoma<br>Normal Adjacent	67	M	T2bM0	G3		Increased
BM-35	Adenocarcinoma<br>Normal Adjacent	67	M	T2bNxM0	G3	II-III	No Change

BM-36	Adenocarcinoma<br>Normal Adjacent	62	M	T2aNxM0	G3	II-III	No Change
